# Fragmented Nuclear DNA Is the Predominant Genetic Material in Human Hair Shafts

**DOI:** 10.3390/genes9120640

**Published:** 2018-12-18

**Authors:** Michael D. Brandhagen, Odile Loreille, Jodi A. Irwin

**Affiliations:** DNA Support Unit, FBI Laboratory, 2501 Investigation Parkway, Quantico, VA 22135, USA; mdbrandhagen@fbi.gov (M.D.B.); oploreille@fbi.gov (O.L.)

**Keywords:** hair shaft, mitochondrial DNA, mtDNA, mtGenome, nuclear DNA, next-generation sequencing

## Abstract

While shed hairs are one of the most commonly encountered evidence types, they are among the most limited in terms of DNA quantity and quality. As a result, nuclear DNA short tandem repeat (STR) profiling is generally unsuccessful and DNA testing of shed hair is instead performed by targeting the mitochondrial DNA control region. Although the high copy number of mitochondrial DNA relative to nuclear DNA routinely permits the recovery of mitochondrial DNA (mtDNA) data in these cases, mtDNA profiles do not offer the discriminatory power of nuclear DNA profiles. In order to better understand the total content and degradation state of DNA in single shed hairs and assess the feasibility of recovering highly discriminatory nuclear DNA data from this common evidence type, high throughput shotgun sequencing was performed on both recently collected and aged (approximately 50-year-old) hair samples. The data reflect trends that have been demonstrated previously with other technologies, namely that mtDNA quantity and quality decrease along the length of the hair shaft. In addition, the shotgun data reveal that nuclear DNA is present in shed hair and surprisingly abundant relative to mitochondrial DNA, even in the most distal fragments. Nuclear DNA comprised, at minimum, 88% of the total human reads in any given sample, and generally more than 95%. Here, we characterize both the nuclear and mitochondrial DNA content of shed hairs and discuss the implications of these data for forensic investigations.

## 1. Introduction

Shed hairs are one of the most commonly encountered evidence types [1], but also among the most limited in terms of DNA quantity and quality. By some estimates, shed hair represents up to 90% of the hair samples collected at crime scenes [2,3]. Unfortunately, nuclear DNA (nuDNA) is generally too low in quantity and quality to permit successful short tandem repeat (STR) typing. 

The difficulty of successful STR typing of shed hairs is attributed primarily to the keratinization process, which degrades cellular organelles and nucleic acids [4]. Not only do the general enzymatic activities associated with keratinization result in DNA degradation, but also nuclear DNA, in particular, is specifically targeted for destruction [5,6,7]. Nevertheless, some nuclear DNA is known to persist in telogen hairs, albeit at low quantities and highly variable qualities [4]. Though a number of studies describe the presence, and successful PCR amplification, of autosomal STR markers from telogen hairs [2,8,9,10,11,12], STR typing is not routinely pursued in forensic laboratories for a number of reasons. For one, reduced size STR amplicons, ranging from ~60–150 bp, are generally required to achieve amplification success. Yet, since only a relatively small number of reduced sized amplicons can be multiplexed with currently employed capillary electrophoresis technologies, data recovery is somewhat limited. Second, elevated cycle numbers, often between 30 and 40, are generally required to amplify the low quantities of nuclear DNA to detectable levels. However, even when reduced size amplicons and elevated cycle numbers are employed, amplification success is still inconsistent, and resulting STR profiles are often incomplete. In addition, commonly employed quantitative PCR (qPCR) assays rarely yield enough information to adequately inform downstream analysis. The qPCR assays routinely implemented are simply not sensitive enough for the low levels of nuclear DNA present in telogen hairs [12]. More recent studies have shown that direct amplification of telogen hairs (i.e., amplification without preliminary DNA extraction), when combined with elevated cycles, can result in full STR profiles approximately 20% of the time [12]. However, it is difficult or impossible to predict a priori if a hair will yield probative DNA data. 

It is also the case that success rates in routine forensic practice are likely lower. Most research studies are necessarily based on freshly, or relatively recently, collected hair samples. While general patterns can obviously be ascertained from such samples, the variability and difficulty of aged and/or damaged casework samples is nearly impossible to accurately represent. Indeed, in those studies for which hairs recovered from actual crime scenes were included, the evidence hairs performed worse than the recently collected hairs [10,13]. Given that amplification success cannot be predicted in advance, and that amplification success rates are generally low, it simply does not make sense from a practical perspective to exhaust limited sample material on a testing modality unlikely to yield probative results. Instead, mitochondrial DNA (mtDNA) is routinely sought in these cases due to its abundance relative to nuclear DNA. Though mtDNA profiles do not offer the discriminatory power of nuclear DNA profiles, mtDNA is recovered in 92.5% of telogen hair cases [14]. 

Because of the difficulty of recovering nuclear DNA from hair, studies conducted to characterize and better understand the state of DNA in hair have largely focused on the more accessible mtDNA molecule. At a broad level, Melton et al., [14] showed that with increasing age of the hair specimen, the likelihood of obtaining a full hypervariable region I/hypervariable region II (HVI/HVII) profile decreased. The same pattern was observed in a systematic study by Gilbert et al. [15]. Additional studies, based on the size of recoverable PCR amplicons, have shown progressive degradation of mtDNA along the hair shaft [16,17,18] with DNA quality deteriorating rapidly within a few millimeters of the root [10]. 

Some of the most recent information on the overall quantity and quality of DNA in telogen hair shafts has come from studies employing next-generation sequencing (NGS). Because NGS-based shotgun sequencing is not dependent on pre-defined amplicons, the sequence data reflect the endogenous size of the DNA. Generally speaking, these studies show that DNA preservation in aged hair is overwhelmingly poor [19,20,21,22]. For example, the average mtDNA size of a 4000-year-old paleo Eskimo sample was 76 bp [19], and mtDNA averaged only 61 bp (range between 48 to 73 bp) [22] in 111 human hairs collected between 1920 and 1970. 

Preliminary studies in our laboratory showed that in shotgun libraries of two freshly collected single shed hairs, 99.93 and 99.88% of the reads mapping to the human genome were nuclear DNA, and the remaining 0.07% and 0.12% were mitochondrial DNA [23]. These results are consistent with those of other shotgun sequencing studies of aged hairs. In particular, Rasmussen et al. [20] found that ~80% of the reads produced from a 1.5 g sample of 4000-year-old hair were human sequences and that only 0.13% of the human reads were mtDNA sequences. The remainder, 99.87%, were nuclear DNA sequences.

Here, we aim to confirm that, despite a high level of degradation, nuclear DNA comprises the vast majority of total human DNA in hair shafts. In addition, we further characterize the quantity and quality of both mitochondrial and nuclear DNA that can be recovered from single shed telogen hairs regularly encountered in forensic casework. 

## 2. Materials and Methods 

All tested samples were rootless hair shafts collected with informed consent from the donors under FBI Institutional Review Board approved project #417-17 (Table 1 and Table 2).

### 2.1. Types of Hair

#### 2.1.1. Recent hairs

The hairs referred to as recent hairs (R series) were cut or collected less than 6 years before the date of the DNA testing and stored primarily at 4 °C. Recent hairs were collected from hairbrushes or by finger combing from random portions of the head. Prior to DNA extraction, the root (proximal) ends of these hairs were cut (~1 cm) and removed. Six recently collected hair samples were used to characterize total DNA (mitochondrial and nuclear) content in rootless shed hairs and approximately 5 to 6.5 cm was extracted for any given hair (Table 2).

#### 2.1.2. Aged Hairs

The aged hairs (A series) were taken from hair cuttings that were ~40 to 60 years old and had been stored at room temperature. Because the samples were hair cuttings, the lengths of the hairs at the time of cutting, and thus the distances from the scalp of the tested segments, were unknown. Furthermore, it was not possible to easily identify the proximal and distal end of these hairs. Samples A1, A2, A6 and A7 were children’s hairs that originated from three individuals whose mtDNA genome (mtGenome) profiles were known. Both hair types were used to evaluate: (1) mtDNA and nuclear DNA content, (2) mtDNA quantity and quality along the length of the shaft, (3) the possibility of complete mtGenome sequence recovery and 4) nuclear DNA quantity and quality along the length of the shaft. 

#### 2.1.3. Segmented Hairs

**Figure 1:**
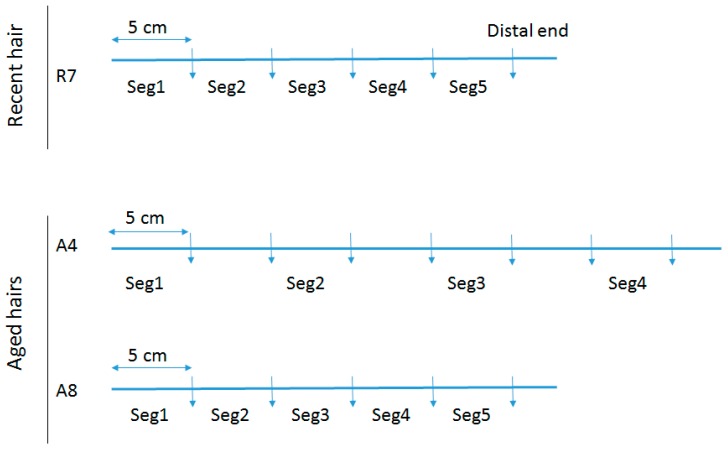
Segmentation of hairs. Hairs R7, A4, and A8 were long hairs cut in segments used to assess total DNA quantity and quality along the length of individual hair shafts.

To assess total DNA quantity and quality along the length of individual hair shafts, a recent untreated hair that had been stored refrigerated for 4 years (R7) and two separate aged hairs (A4 & A8) were tested. Following removal of the root (if necessary), the hairs were cut into 5 cm segments. For R7, the mtGenome profile of the donor was known and five segments were tested (see Figure 1), with Segment 1 representing the segment closest to the root end. For A4, seven segments were cut but only four were tested. For A8, five continuous segments were tested. Samples A4 and A8 are from a >50 cm adult braid that was cut at least 40 years ago and for which no reference profile was available. 

### 2.2. Extraction

**Figure 2:**
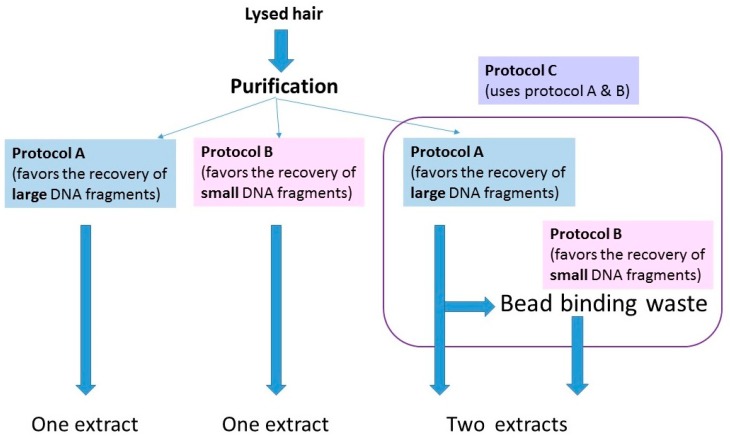
Schematic representation of the three purification methods used in this study. For details on the protocols, see Protocols, in the Supplementary Materials.

The protocols for washing and digesting the hair samples are presented in Protocols, Supplementary Materials. Once the hairs were fully digested, lysates were purified with one of three protocols (Figure 2). The purification methods were being tested for a different study, but essentially served as replicate extractions for those hairs purified with multiple protocols. The protocols are summarized below:

(1) A protocol adapted from a Qiagen user-developed method [24,25]) that has been employed in the FBI Laboratory’s routine casework since 2014 (referred to from here on as protocol A; see Protocols in the Supplementary Materials).
(2) A protocol based on Allentoft et al., [26] that employs a binding buffer that favors the recovery of small (<100 bp) DNA fragments.
(3) A combined protocol that follows protocol A until the step at which the lysate and silica beads are on the magnet. For this protocol, (protocol C in Protocols, Supplementary Materials), the bead binding waste solution was retained and mixed with the binding buffer from protocol B. It was subsequently purified and eluted with MinElute columns (Qiagen, Germantown, MD, USA).

Table 2 summarizes the purification protocol used for each hair sample tested in this study. Generally speaking, protocol A was used for the purification of recently collected hairs while protocol B was used with aged hairs for which DNA was expected to be degraded.

### 2.3. Quantification

Quantity of mtDNA was assessed by qPCR of the DNA extracts. For the recent hair extracts, quality of the mtDNA was assessed in one of two ways: by a mtDNA qPCR assay developed by Kavlick [27] on a 7500 Real Time PCR system (Thermo Fisher Scientific, Waltham, MA, USA) or by sequence read length for the aged hair extracts. 

For the mtDNA quantification, two microliters of each extract and reagent blank (RB) were amplified in duplicate with a qPCR assay that incorporates a DNA degradation index. With this assay, degradation is assessed based on the ratio of large (≥316 bp) and small (≥105 bp) mtDNA fragments. The larger the degradation index, the lower the number of mtDNA fragments ≥316 bp relative to fragments between 105 bp–316 bp. For example, a degradation index of 1 or lower indicates no mtDNA degradation (all quantified fragments ≥316 bp), while a degradation index >1 indicates that fewer 316 bp or larger fragments are present in the extract than 105 bp–316 bp fragments [27]. An undetermined degradation index indicates that all the human mtDNA is degraded to a size smaller than 316 bp.

Sequence read lengths could not be used to assess endogenous mtDNA quality in the recent hair extracts because, following qPCR, the DNA required shearing to ensure successful library preparation and sequencing. This is due to the presence of large mtDNA fragments that have been shown to exist in freshly collected hair shafts [23]. Conversely, qPCR could not be used to assess mtDNA quality in the aged hairs because the degradation index, which is dependent on the amplification of DNA fragments of 105 and 316 bp in size, could not be recovered from most of the aged hair segments.

Nuclear DNA quantification of the recent hair extracts using the Quantifiler Trio DNA quantification kit (Thermo Fisher Scientific) was attempted for a number of samples, but the results were too low to be useful (<0.5 pg/µL).

### 2.4. Library Preparation

Following quantitation, aged hair DNA extracts were used for library preparation. For recent hairs, to ensure successful downstream library preparation and sequencing, DNA was fragmented prior to library preparation. Shearing was performed with the Fragmentase enzyme present in the KAPA HyperPlus Library Preparation Kit (Kapa Biosystems, Wilmington, MA, USA) for 30 min. Extracts were then purified with a Qiagen MinElute PCR purification kit, and eluted in 50 µL of H_2_O.

50 µL of each extract or RB was then converted to an Illumina library using the NEBNext Ultra II kit (NEB, Ipswich, MA, USA) and looped adapters from the NEBNext Multiplex oligos for Illumina kit (NEB). The libraries of the aged hairs were prepared according to the manufacturer’s instructions with the exception of the ligation which was performed overnight at 7 °C. Following ligation, the looped adapters were converted into Y-shaped adapters and the libraries purified with Ampure XP beads (Beckman coulter, Sykesville, MD, USA). All libraries were dual-indexed with indexed primers from the NEBNext Multiplex oligos for Illumina kit and subsequently amplified for 25 cycles with the NEBNext Ultra II Q5 PCR kit. Purification was performed using Ampure XP beads.

### 2.5. Sequencing

All libraries were shotgun sequenced on an Illumina MiSeq FGX instrument with a 300 cycles v2 cartridge and 2 × 150 cycles + 2 × 8 cycles for the indexes for the aged hairs and a v3 2 × 300 cycles + 2 × 8 cycles for the recent hairs.

### 2.6. Data Analysis

Read mapping and consensus variant calling were performed with the CLC Genomics Workbench software, version 10.0.1 (CLC Bio, Qiagen). All reads were trimmed and overlapping pairs merged (see details in CLC workflow, Supplementary Materials). The default Genomics Workbench mapping and alignment parameters, which included a length fraction of 85% and a similarity fraction of 97%, as well as insertion/deletion (indel) and mismatch costs of 3, were used for all samples. Alignments to the mtGenome were performed using the revised Cambridge Reference Sequence (rCRS, [28]). MtGenome variant calling was performed using the Fixed Ploidy variant caller.

Alignments to the human genome were performed using the human reference genome sequence build hg38. The percentages of reads mapping to both the mitochondrial and nuclear genomes were determined based on summary data and mapping statistics produced by the CLC software.

## 3. Results

Comprehensive overviews of all data can be found in Tables S1–S3, Supplementary Materials.

### 3.1. Mitochondrial DNA in Recent Hairs

To assess the quantity and quality of DNA in recent hair, five 5 cm segments of a single recent hair shaft (R7) were tested with two purification methods that served as replicates (Protocol A and Protocol B) and then quantified via qPCR (Table 3 and to see mtDNA copies/ml (see Table S1, Supplementary Materials). The absolute quantity of mtDNA recovered from any given hair segment differed based on the purification method used. Protocol A, which targets DNA fragments generally larger than 100 bp, yielded between 1.5 and 5.4 times more mtDNA fragments than protocol B for any given hair segment. However, the overall patterns of mtDNA quantity and quality observed within a single hair were the same regardless of DNA purification method. 

Results from both purification methods showed that segments more distal to the root contained lower mtDNA quantities than segments more proximal to the root (Figure 1). For protocol A, the quantity of mtDNA fragments in the segment closest to the root (1 to 6 cm from root, as the first 1 cm with root had been removed) was 43,992 mtG/cm, while the quantity of mtDNA fragments in the most distal segment (approximately 21–26 cm from root) was 10,296 mtG/cm (Table 3). Overall, a 4-fold decrease in DNA quantity was observed along the length of the hair. Similarly, for protocol B, the quantity of mtDNA fragments in the segment most proximal to the root was 17,261 mtG/cm, with only 2802 mtG/cm recovered from the segment 21–26 cm from the root. 

This trend in the reduction in quantity of mtDNA moving from the proximal to the distal end of the hair was also observed in the sequencing data of the protocol B DNA extracts (Table 4). Not only did the number of mtDNA reads decrease from 4255 to 738 along the length of the shaft, but also the percentage of mtGenome data recovered declined (Table 4 and Table 5). At a read depth of 2x, coverage of 99.94% of the mtGenome was recovered at the proximal end, but only 42.27% and 55.88% at the distal ends. Similarly, at a read depth of 10x, mtGenome coverage fell from 80.16% to 4.97%. 

Regardless of the purification method used, the declines in quantity generally followed a linear pattern (Table 3). However, with both purification protocols, the most distal segment reflected more mtDNA than the segment immediately proximal. While this could reflect a true increase in DNA quantity in the distal segment of the tested hair (as the increase is seen in both purification replicates and the replicates were created after hair lysis), something about the particular hair segment or lysate would seem to explain the results (e.g., less efficient binding to silica, stochastic amplification, etc.) as the DNA degradation indices and average fragment sizes of the distal segments did not follow the same pattern. Overall, and depending on purification method, between four and six times less DNA was recovered from the distal segments than the proximal segments. 

The two replicates, or purification methods, also revealed consistent patterns when DNA quality was assessed. For protocol A, a degradation index (DI) of 1.53 was obtained from the Segment 1 extract, and the DI increased to 3.44 in Segment 5 (Table 3). For protocol B, the degradation factor increased from 1.50 to 3.69. In both cases, and similar to the reduction in mtDNA quantity along the length of the shaft, the degradation indices suggested a consistent reduction in the quality of mtDNA along the length of the shaft. 

**Figure 3:**
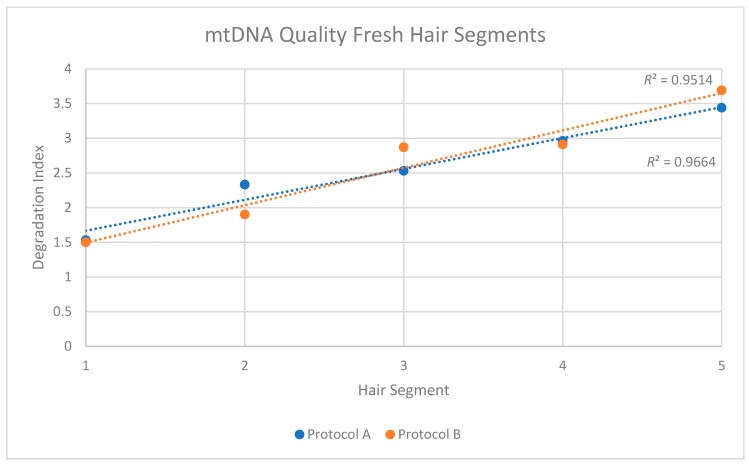
Mitochondrial DNA quality in five segments of recently collected hair. Two replicates (orange series and blue series) were performed for each segment.

When the individual DIs from each replicate were plotted, *R*^2^ values of 0.9514 and 0.96641 were obtained for Protocols A and B, respectively (Figure 3). In both cases, the trend was not only consistent along the length of the shaft, but also statistically significant (Replicate 1: *p* < 0.05; Replicate 2: *p* < 0.05). This reduction in quality was confirmed in the sequencing data from protocol B DNA extracts of the hair segments (DNA was not fragmented before sequencing in these cases). The data showed that the average mtDNA size decreased from 168 bp at the proximal end to 91 bp at the distal end (Table 4).

The potential of recovering complete mtGenome sequence from shotgun sequence data of recently collected hairs was tested using 5 cm–6 cm of six rootless hairs (R1–R6, Table 2). Cuttings from hairs R1, R3, R4, R5, and R6 were each composed of the first 1–6 cm proximal segment of an approximately 20–25 cm length hair. R2 was composed of several ~1.3 cm cuttings from the most distal portion of several ~5 cm hairs (from a haircut). The six DNA extracts varied in mtDNA quantity between 400 mtG/cm to 103,181 mtG/cm and reflected degradation indices ranging from 1.93–5.86 (Table 6). The quantity and quality values appeared to show no correspondence to hair treatment or time of storage. The differences may be due simply to the variability known to exist between hairs [16,18]. Despite the wide variation in mtDNA quantity and quality of the various hair shafts tested, complete mtGenome sequences were recovered from five of the six samples with at least 2x coverage. Depths of coverage in these cases averaged 44x and ranged between 2x and 844x (Table 6). The sixth sample (R2), which was also the sample with the lowest mtDNA yield, produced data covering approximately 73% of the mtGenome (Table 6). In this case, the average read coverage was 10x, with a minimum of 0 reads and a maximum of 163 reads. In an attempt to recover DNA fragments that may be lost during Protocol A extraction from recent hairs R1–R3, the bead binding waste solution was retained and purified/extracted with the binding buffer from Protocol B (Protocol C). An additional 5902 mtG/cm for R1, 13,410 mtG/cm for R2, and 11,765 mtG/cm for R3 were recovered.

### 3.2. Mitochondrial DNA in Aged Hairs

To better understand total mtDNA quantity and quality in the types of degraded hair samples often encountered in forensic casework, four aged hair samples were evaluated via both mtDNA qPCR and sequencing (Table 7). The hairs had been cut in 1962 (A1, A6), in 1958 (A2), and in 1978 (A7) and stored at room temperature. Based on quantitative PCR data from Table 7, the samples exhibited mtDNA yields substantially lower than the yields from the recent hair samples, even when quantitation values from the aged hairs were compared to the two most distal segments of the recently collected hair (17–22 and 22–27 cm from the root; quantitation values ranged between 1414 and 10,296 mtG/cm). As with the recent hairs, the purification method used on the lysates had a noticeable effect on the absolute quantity of mtDNA recovered (only protocol B used for A6 and A7). In the case of the aged hairs A1 and A2, however, the protocol B extracts—which targeted smaller fragments—yielded more DNA than the protocol A extracts. For A1, mtDNA yields were 20 mtG/cm for protocol A and 100 mtG/cm for protocol B, while for A2 the mtDNA yields were, respectively 67 mtG/cm and 947 mtG/cm. Though based on only a handful of data points, the lower calculated mtDNA quantities (as measured by the 105 bp qPCR molecule) but recoverable degradation indices (which reflects amplification of at least some ≥ 316 bp fragments) for only protocol A extracts is likely due to the preferential recovery of larger DNA fragments with protocol A. Protocol B, which preferentially recovers smaller DNA fragments, consistently yielded higher quantitation values, but no degradation values.

The increased recovery of smaller DNA fragments from protocol B was directly observed in the sequencing results. Depending on the hair, between 58 and 309 times more sequencing reads were recovered from the protocol B extracts than the protocol A extracts, resulting in only 0–8.28% and 2.12–36.54% mtGenome coverage with Protocol A and 98.93–100% mtGenome coverage with protocol B (Table 7). This suggests that the majority of DNA in the aged hairs was extremely fragmented and lost with purification protocol A. Regardless of this recovery difference, however, the degradation state of the DNA as indicated by both purification methods was consistent. For A2 (cut and collected in 1958), the average size of the mtDNA reads from protocols A and B were 87 bp and 70 bp, respectively (Table S3 in Supplementary Materials). For A1 (cut and collected in 1965), the mtDNA reads averaged 71 and 55 bp.

Because of the substantially improved data recovery from the aged hairs with protocol B, mtDNA quantity and quality along the length of the shaft was assessed with protocol B only. For this evaluation, DNA was extracted from four, 5 cm segments of a single ~40-year-old hair shaft (Seg 1 to 4-A4*) and then assessed via both qPCR and sequencing. Similar to the results from the recently collected hairs, the qPCR results from the aged hairs showed a consistent decrease in mtDNA quantity as segments more distal to the root were tested (Table 8). While the most proximal segment (1–6 cm from the root) yielded 3330 mtG/cm, the most distal segment (31–36 cm from the root) yielded only 510 mtG/cm (Table 8). 

**Figure 4:**
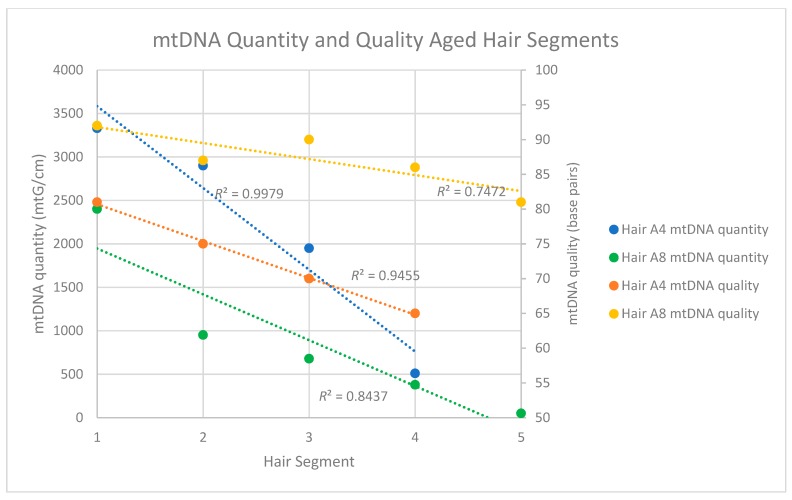
Mitochondrial DNA quality and quantity measures for two single, aged segmented hairs. For hair A4*, the four 5 cm segments spanned 35 cm of hair. For A8, the five 5 cm segments spanned 25 cm of hair. Both came from the same donor.

Degradation indices were not recovered from any of the aged hair segments, most likely due to the fact that DNA fragments of the size required to derive the degradation index (≥316 bp) were simply not present in the tested segments. MtDNA quality was instead assessed based on the read lengths of the shotgun sequencing data. Again, similar to the recent hairs, a reduction in mtDNA fragment size was observed as segments from the proximal to distal end of the hair were tested (Table S3, Supplementary Materials). MtDNA read lengths (and, by proxy, endogenous mtDNA fragment size) in the most proximal segment of the hair averaged 81 bp, with read lengths decreasing to an average of 69 bp in the most distal fragment (Table 13). When mtDNA quantity and quality were plotted against hair segment for Sample A4, *R*^2^ values of 0.9455 and 0.9951 were obtained for mtDNA quantity and quality, respectively (Figure 4). In both cases, the trends were not only very consistent along the length of the shaft, but also statistically significant (Quantity: *p* < 0.05; Quality: *p* < 0.05). A similarly consistent trend was observed with the mtDNA quantities of the five segments of hair A8*, but not necessarily with the mtDNA qualities. Though the average size of the most distal A8* segment was smaller than the most proximal end, the decline was not consistent from segment to segment (Figure 4, Table 13). This may be a result of the fact that the A8* segments spanned a shorter length of hair than the A4* segments (see Figure 1). 

In terms of complete mtGenome sequence recovery, and not surprisingly given the observed mtDNA quantities and degradation states, the aged hairs were less successful in producing full mtGenome data than the recent hairs (Table 7). The aforementioned difference in DNA yields from the two different purification protocols made a clear difference in the recovery of complete mtGenome data. Only two aged hairs were tested with both protocols (A1 and A2), and complete mtGenome data were recovered only from the lysates purified with protocol B. More than likely, the small endogenous DNA fragments were simply washed away with protocol A, leaving close to nothing to be sequenced in these extracts. In the two cases for which sequencing was successful, complete mtGenomes were recovered despite low quantification values (100 mtG/cm for A1* and 947 mtG/cm for A2*). For A1*, the complete genome sequence was based on 31,762 unique reads and an average coverage of 92x, while the A2* genome was based on 44,826 unique reads and an average coverage of 84x (Table 7 and Table 9). 

Though A1* and A2* yielded 100% mtGenome coverage at 10 reads, and A6* & A7* yielded ~99% coverage, the segments of aged hairs A4* and A8* yielded less (Table 10). At a depth of 10 reads, zero to 15% of the genome was covered for all segment extracts except Seg1-A8 (93.6%). At a depth of coverage (DOC) of five reads, however, approximately half of the genome was covered for all samples (except Seg1-A8 at 99.05%), and at a depth of two reads, with the exception of Seg5-A8*, over 80% of the genome was covered for all samples (Table 10). Interestingly, even in shotgun data, the control region generally had the highest DOC compared to the rest of the mtGenome. This may be due to the GC content of the CR (Figure S1, Supplementary Materials). In all cases for which the mtDNA profile of the donor was known, the sequence data corresponded to the known profile.

### 3.3. Nuclear DNA from Recent Hairs

The potential of nuclear DNA recovery from rootless shed hairs was assessed with six recently collected hairs (R1-R6). For all hairs, nuclear DNA quantification values could not be recovered via traditional nuclear qPCR, and thus nuclear DNA content was assessed based solely on the ratio of nuclear to mitochondrial sequence reads in the final data. For these six recently collected samples, between 34,909 and 952,728 unique reads mapped to the human genome. Of these, 33,044 to 927,153 mapped to the human nuclear genome, with the remainder in any given sample mapping to the human mitochondrial genome (Table 11). In total, the percentage of reads mapping to the human nuclear genome for any particular recently collected hair ranged between 88.4–99.5% of the total DNA reads (Table 11).

### 3.4. Nuclear DNA in Aged Hairs

For almost all of the aged hairs, more than 99% of the shotgun reads that mapped to the human genome were nuclear DNA sequences (between 99.1 and 99.9%) while the remaining 0.1–0.9% mapped to the mtGenome. Sample A7 had 92.3% align to nuDNA and 7.7% align to mtDNA. These high percentages of nuclear DNA were observed regardless of the purification protocol (both of which were used for the assessment of A1 and A2). The average size of the nuclear DNA reads varied between 49 and 88 bp, and for five of the six extracts, the average read length of the nuclear DNA fragments was smaller than the average read length of the mtDNA fragments from the same extract (Table 12). For sample A2*, the mtDNA fragments averaged 69 bp in length, while the nuclear DNA fragments averaged only 57 bp. The same trend held true for samples A2 and A1* where the mtDNA fragments averaged, respectively, 87 bp and 55 bp and the nuclear DNA fragments averaged 58 bp and 54 bp. The only exception to this pattern was sample A1, for which mtDNA reads averaged 71 bp, while nuclear DNA reads averaged 88 bp. This result could be authentic, or, given the small number of mtDNA reads (145) upon which the read length average is based, the larger size of the recovered nuclear DNA fragments could simply be the result of read sampling. Given that the four segments of aged hair (samples A4 Seg1–Seg4 and A8 Seg1–Seg5) also showed a pattern of the nuclear DNA being smaller than the mitochondrial DNA, it seems likely that the sample A1 results are a sampling issue (Figure 4).

**Figure 5:**
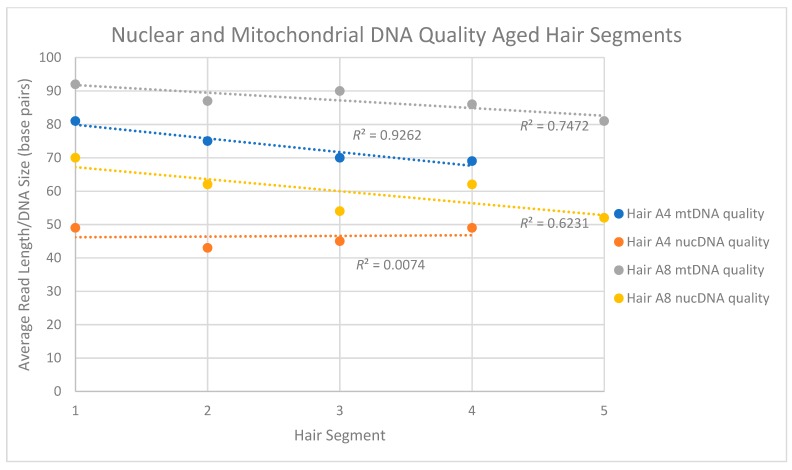
Nuclear and mitochondrial DNA quality (i.e., read length) for segments of two aged hairs. For hair A4*, the four 5 cm segments spanned 35 cm of hair. For A8*, the five 5 cm segments spanned 25 cm of hair. For each segment, and regardless of the hair sample, the average size of the mtDNA fragments was larger than the average size of the nuclear DNA fragments. In addition, with the exception of the hair A4 nuclear DNA, fragment sizes tended to decrease along the length of the hair shaft.

For the four aged hair shaft segments Seg-A4*, nuclear DNA read lengths ranged between 43 and 49 bp while mtDNA fragments averaged between 69 and 81 bp. Interestingly, however, while the mtDNA read length seemed to consistently decline along the length of the hair shaft (Figure 5) there appeared to be no clear pattern of a decrease in quality (i.e., size) for the nuclear DNA. Five segments from a second aged hair (Seg-A8*) did not show a similar consistent decline in mtDNA read length but did repeat the overall trend of the mtDNA fragments (81–92 bp) being larger than nuDNA fragments (52 to 70 bp) in any given segment. For both sets of samples, the total nuclear DNA content mirrored observations from the other recently collected and aged hair samples, with between 94.3 and 98.9% of the human DNA reads mapping to the nuclear DNA genome (Table 13). 

## 4. Discussion

### 4.1. Mitochondrial DNA Content in Single Shed Hairs

Because of the difficulty of recovering nuclear DNA from shed hair, what is known about the DNA content of single shed hair comes primarily from studies of mitochondrial DNA. Studies have shown the mtDNA content of shed hairs to be highly variable in quantity and quality both within and between single hairs [4,13,18,29,30]. In addition to this natural variability are the additional factors routinely encountered in a forensic context: the age of the hair specimen, the environment from which it was recovered, and/or the chemical or physical insults to which the sample may have been subjected. The success of mtDNA profile recovery is heavily dependent on such factors [14]. Though we do not directly address the variation in mtDNA content between different individuals or between single hairs from the same individual in the present study, our results are directly in line with other reports regarding the quantity of mtDNA within single hairs [18,29]. For recently collected hairs, our results show a consistent, approximately 4-fold decrease along the 25 cm of the hair shaft. With the aged hairs, an approximately 6.5-fold decrease was observed along the 35 cm tested. These are smaller decreases than have been observed in earlier studies [18,29]. However, the numbers are difficult to directly compare given the high variability among hairs, different hair sampling strategies and sample numbers, and different approaches for quantifying the mtDNA (i.e., size of the amplicon). In addition, it is clear from the data reported here that extraction protocols themselves can impact DNA recovery and perceived DNA quantity. Regardless of these differences, the picture remains the same: mtDNA quantity decreases substantially along the length of the shaft.

In addition to confirming earlier observations of decreasing quantities of mtDNA along the length of the shaft, we also observed decreasing quality along individual hairs. It is generally assumed that as the quantity of DNA decreases along the length of the hair shaft (or perhaps because the quantity decreases along the shaft), the quality of DNA also decreases. Yet, there are actually very few empirical data that directly address the question. The few studies that have been performed have generally addressed the question by targeting individual amplicons of various sizes within hair shaft segments [18] and demonstrating, as expected, that quality decreases. We addressed the question in two ways. With the recently collected hair, and similar to the approach used by others, we assessed mtDNA quality via the degradation index produced by qPCR. In this case, the degradation index of the hair consistently increased from approximately 1.5 to approximately 3.5, indicating a 3-fold shift (increase) in the quantity of the smaller sized amplicon relative to the larger sized amplicon. This shift has, in previous studies, been shown to result in an inability to recover complete mtDNA control region or the mtGenome using larger amplicon primer sets [18]. Shotgun sequence data from the recent hair segment extracts confirmed this trend as the mtDNA averaged 79.87 bp in size at the proximal end of the shaft and then decreased systematically to 43.89 bp at the distal end—25 cm away. For the aged hairs, the degradation index of the qPCR could not be used to assess quality because the sizes of the endogenous DNA fragments were generally not large enough to yield qPCR product. Nevertheless, the pattern of decreasing mtDNA fragment size along the length of the shaft was still clearly evident from the shotgun sequence data. This fine-scale view of the data from sample A4* showed that the mtDNA averaged 81 bp in size at the proximal end of the shaft and then decreased systematically to 69 bp at the distal end, 30 cm away. 

Given the extremely small size of the mtDNA fragments recovered from the aged hairs, it is not surprising that probative mtDNA data are more difficult to recover in forensic cases involving older, and damaged or degraded hair samples. Melton et al., [14] describe that while over 90% of hair samples aged 20 years and younger produce some mtDNA data using standard Sanger sequencing techniques, successful mtDNA recovery drops to 60% of samples aged 30 years or older. Not only are the DNA extraction and purification protocols routinely employed in forensic laboratories not optimized for the recovery of fragments <50 bp in size, but also the vast majority of downstream DNA typing assays are designed for DNA fragments >100 bp [13,31,32]. Thus, even if the extremely small fragments recovered from the aged samples tested here were routinely recovered at the extraction stage, DNA typing via traditional targeted PCR would still be unsuccessful. These factors are also likely why complete control region sequences could only be recovered in approximately 50% of hair segments tested in Desmyter et al., [18] where the decline in sequence recovery corresponded with the decline in mtDNA quantity towards the distal ends of the hairs.

For all single recent hairs but one (R2), the shotgun sequencing strategy employed in the current study, produced between 98 to 100% of the mtGenome (DOC of 5x), regardless of mtDNA quantity or quality. For the incomplete sample, 55% of the genome, was still recovered with a DOC of 5x. Interestingly, the single recently collected hair sample that yielded incomplete data behaved, by all measures, more like the aged hair samples than the recent hair samples. Not only did it yield very little DNA when the purification protocol favoring larger DNA fragments was used (unlike the other recent hairs), it also showed levels of DNA damage that exceeded levels observed in the aged hairs. It is possible that these observations demonstrate the extreme variability in hairs between individuals [14,16,18].

We have previously reported the recovery of complete mtGenome sequence information from shotgun data of two recent shed hairs [23]. In those cases, the hair fragments tested were immediately proximal to the root, included the root bulb (but no tissue), and were approximately 10 cm long. Here, we have extended those findings by demonstrating that complete mtGenome sequences can be recovered using shotgun sequencing from smaller hair fragments (2.5 cm versus 10 cm) and fragments that do not include the root end. Given that qPCR quantification and degradation index values could be recovered from most of the recent hairs, it is likely that mtDNA fragments of a size sufficient for targeted PCR amplification are present, and that commercial assays targeting small amplicons could also be successful in producing complete mtGenome data. 

The same is likely not true for the aged hair samples, however. In the case of the hairs tested here, mtDNA fragment sizes averaged only between 55 and 87 bp, depending on the sample. This is consistent with other NGS-based studies on mtDNA from clumps of shed hair that found DNA fragments of approximately 61 bp in samples between 50 and 100 years old [22]. In all cases, these sizes are approximately 100 bp smaller than the average amplicon size in commercially available mtDNA assays [33]. Nevertheless, in all of the single hair shafts tested here (unsegmented rootless hairs greater than 40 years old), complete or near complete mtGenomes were recovered even when no mtDNA quant values were produced. While we have shown here that complete mtGenomes can be recovered from extremely old hair even without enrichment, hybridization capture assays that enrich for mtDNA would almost certainly improve efficiency. Indeed, when several recent hair segments and a number of aged hairs not included in this study were enriched for mtDNA with a hybridization capture assay, complete mtGenomes were routinely recovered (Table S4 in Supplementary Materials). 

### 4.2. Nuclear DNA Content in Single Shed Hairs

With the recovery of complete mtGenomes, we were also interested in the potential to both recover and characterize the nuclear DNA content of single shed hairs. Though autosomal short tandem repeat profiles have been shown to be recoverable from such samples, success rates for producing informative profiles (e.g., 8 loci or greater) are understood to be low [8,9,10,11,12,34]. As a result, it is generally assumed that nuclear DNA is simply not present in high enough copy number and/or is too degraded to be recovered in shed hairs. In our own laboratory, attempts to recover STR profiles from shed telogen hairs using the capillary electrophoresis-based protocols routinely employed in operational casework produced full STR profiles in only 4.4% of the samples tested; and both of these samples (out of 45 total) included the root end. 

Unfortunately, because of the library preparation for the recent hair, which necessarily required DNA shearing to permit successful downstream sequencing, the native fragmentation state of the nuclear DNA could not be assessed in the recent samples. However, data from the aged hairs showed extreme degradation, with average nuclear DNA fragment sizes of just 43 bp–88 bp sizes far smaller than those required for targeted PCR amplification with the typical commercially available assays. The nuclear DNA was also consistently found to be more heavily fragmented than the mtDNA. In all but one of the extracts tested, the average nuclear DNA length was smaller than the average mtDNA length, with the only exception to this pattern likely a data sampling issue. It should be noted here that the purification protocol employed had a clear and direct impact on the absolute length of nuclear and mtDNA fragment sizes recovered (with smaller average sizes observed for both the mtDNA and nuclear DNA fragments when the protocol favoring smaller fragments—B- was employed). However, the length of the nuclear relative to the mitochondrial DNA was the same regardless of the purification protocol. In both cases, the average mtDNA fragment size was consistently larger than the average nuclear DNA fragment size.

Though the nuclear DNA was found to be more degraded than the mtDNA, it was found in far higher quantity in any given hair or hair sample. In fact, for all of the hairs or hair segments tested, nuclear DNA comprised the vast majority of the human reads (Table S3, Supplementary Materials). This was the case regardless of the sample’s age (recently collected or >50 years old) or the proximity of the tested hair segment to the root. For the six recent hairs tested, and in line with previous studies on freshly collected single shed hair [23], nuclear DNA reads comprised, at minimum, 88% of the total human reads, and generally more than 95%. For the aged hairs, the nuclear DNA read content hovered around 94–99% for the hair segments (samples A4* and A8*), and all but one of the aged hairs reflected nuclear DNA contents of >99%. Nuclear DNA recovery has been reported previously for hair samples of 20 mg to 2 g in weight [19,20,21,22]. The findings here come from less than 1 mg of sample material.

Because of the size of the nuclear genome, the overwhelmingly large percentage of nuclear DNA reads was not sufficient to provide any reasonable depth of coverage across the genome. This was in contrast to the mtDNA data where the relatively small percentage of reads was often adequate to provide complete mtGenome coverage at an average read depth >10 when all samples were considered. Again, these data were produced with an Illumina MiSeq FGx—a low throughput/output instrument, relatively speaking. A higher throughput sequencer (such as a NextSeq or a HiSeq) would likely allow for a substantial improvement in the recovery of both mtDNA and nuclear DNA data. Experiments with these instruments are ongoing. Regardless, a preliminary analysis of 1,201 Y-SNPs based on very low coverage of the nuclear genome with the MiSeq was still sufficient to provide a Y-chromosome haplogroup prediction for hair A2* (Table S5 in Supplementary Materials).

### 4.3. Forensic Applications

A number of commercially available assays are available for next-generation sequencing of forensically relevant markers [33,35,36]. However, all of these assays are based on targeted PCR amplicons. In some cases, the amplicons are as small as 70 bp [37], but generally they are larger than 100 bp. As a result, their utility for samples harboring heavily degraded DNA, such as shed hairs, is limited. 

The utility of NGS approaches that do not require targeted PCR amplification is becoming increasingly recognized in forensics, as it is only with such approaches that DNA from the most degraded specimens can be characterized. Though most applications to date have focused on mitochondrial genome typing from old skeletal remains [38,39,40], a handful of recent studies have also described the recovery of nuclear DNA data from other types of limited and degraded specimens [23,41,42]. The results of these studies, together with the data reported here describing the high content but low quality of nuclear DNA in shed hair, support the hypothesis that a key factor, perhaps *the* key factor, in the inability to recover common STR markers from such samples is the degradation state of the DNA. DNA degradation has long been understood to be a factor in STR recovery from shed hair, with mini-amplicon approaches often proving more successful than standard approaches. However, the persistence and availability of nuclear DNA has also been a question. Studies have suggested that because keratinization involves the breakdown of the nucleus, including the DNA, nuclear DNA is, by extension, simply not present in telogen hair shafts [4,5]. Our data suggest otherwise. Nuclear DNA is present in high quantity, but in extremely small fragments. 

To get a sense of the type of forensically relevant data that could be gleaned from nuclear DNA recovered from rootless shed hair, data from one of the hair samples (A2*) were mapped against the 163 ancestry/phenotype and 104 identity single nucleotide polymorphisms (SNPs) included in the Verogen ForenSeq assay [36]. SNP genotypes were developed for 11 and 14 SNPs, respectively, albeit at low coverage. However, when preliminary tests of a hybridization capture assay that targets those same SNPs were performed, SNP recovery increased to 104/163 for the ancestry/phenotype SNPs and 69/104 for the identity SNPs. Even assuming dropout at all loci exhibiting just one allele, the identity SNP data resulted in a random match probability of one in 10^14^ (the calculation also assumed independence of all SNPs). Though more developmental work will be required to optimize the assay to meet strict forensic guidelines for profile accuracy and reliability, the recovery of nuclear DNA from single telogen hair fragments of less than 1 mg and that yield neither nuclear nor mtDNA quantitation values is promising. 

The fact that DNA data was most effectively recovered from the aged hair specimens when a purification step that preferentially targets small fragments suggests that the DNA of interest in many forensic cases may be inadvertently eliminated at the DNA extraction step. For these types of highly degraded specimens for which routine STR typing is simply not possible due to the quality of endogenous DNA, our results suggest that modified extraction protocols that preferentially recover smaller over larger fragments—and, therefore, recover rather than wash away the target DNA—can be critical to the recovery of probative DNA data in such cases. For most forensic casework, standard extraction protocols that recover DNA fragments of the size required for autosomal STR typing are sufficient. However, if and when the recovery of nuclear DNA is important, and DNA fragment sizes required for STR typing no longer exist, extraction protocols that specifically target smaller fragments may be preferable. In addition, a combination of protocol types, as demonstrated here with Protocol C, may be a viable method to recover both large and small DNA fragments from the same sample.

## 5. Conclusions

In an effort to directly characterize the quantity and quality of mtDNA and nuclear DNA present in the types of limited, aged, and degraded shed hair specimens often encountered in forensic casework, shotgun sequencing was performed on rootless telogen hairs. The results, based on direct observation of the endogenous molecules, revealed that:

1. mtDNA quantity and quality decline along the length of the hair shaft,
2. mtDNA fragments are generally larger than nuclear DNA fragments in the same hair or hair segment,
3. complete mtGenomes can be recovered from aged hair shafts with shotgun sequencing data along (i.e., no enrichment)
4. nuDNA quality tends to decrease along the length of the hair shaft,
5. both nuclear and mitochondrial DNA fragment sizes in the aged hairs were generally <80 bp (too small for routinely employed targeted PCR amplicons) and
6. nuclear DNA was not only recovered but comprised the vast majority of DNA in any given hair sample.

Clearly, the relative sizes and copy numbers of the two genomes play a critical role in the recovery of informative DNA profiles from telogen hair samples (for reference, the percentage of nuclear DNA in a cell with 1000 mtGenomes is 99.997%), but our results show that in the types of specimens that have historically failed to yield mtDNA control region data with existing Sanger protocols, not only could complete mitochondrial genomes be developed, but also informative nuclear DNA data could be recovered. Further development of assays that accommodate the small size of the nuclear DNA may allow for more routine recovery of discriminatory nuclear DNA profiles from such samples. A better understanding of DNA quantity and quality, for nuclear DNA in particular, should promote further development of cost-effective forensic assays that can generate more discriminatory information from not only hair, but also other samples harboring extremely degraded DNA.

## Figures and Tables

**Table 1:** Description of donors and hair samples used in this study. RT = Room Temperature (20–25 °C). § Refers to permanent hair coloring.

Donors	Characteristics	Time between Collection/Cut and DNA Analysis	Temperature Storage	Treated
I	Adult, female, recent	4 years	4 °C	No
II	Adult, male, recent	2 months	RT	No
II	Child, male, aged	53 years	RT	No
III	Adult, female, recent	2 months	4 °C	§Yes
IV	Adult, female, recent	2.5 years	4 °C	No
V	Adult, female, recent	1.5 year	4 °C	No
VI	Adult, female, recent	1 h	RT	§Yes
VII	Child, male, aged	60 years	RT	No
VIII	Adult, female, aged	>40 years	RT	No
IX	Child, female, aged	30 years	RT	No

**Table 2:** Description of each hair sample. The asterisk denotes extracts that were obtained with purification protocol B.

**Recent Hairs**				
**Donor**	**Sample Names**	**Hair Portions**	**Hair Size (cm)**	**Purification Protocol**
I	R1	single hair	5	A
	R1*eluate			C
II	R2	five 1.3 cm hairs	6.5	A
	R2*eluate			C
III	R3	single hair	5	A
	R3* eluate			C
IV	R4	single hair	5	A
V	R5	single hair	5	A
VI	R6*	single hair	6	B
I	Seg1-R7	segment 1	5	A
	Seg1-R7*			B
	Seg2-R7	segment 2	5	A
	Seg2-R7*			B
	Seg3-R7	segment 3	5	A
	Seg3-R7*			B
	Seg4-R7	segment 4	5	A
	Seg4-R7*			B
	Seg5-R7	segment 5	5	A
	Seg5-R7*			B
**Aged Hairs**				
II	A1	two 2.5 cm hairs	5	A
	A1*			B
II	A6*	five 1.5 cm hairs	7.5	B
VII	A2	three 2.5 cm hairs	7.5	A
	A2*			B
VIII	A7*	three 2.5 cm hairs	7.5	B
IX	Seg1-A4*	segment 1	5	B
	Seg2-A4*	segment 2	5	B
	Seg3-A4*	segment 3	5	B
	Seg4-A4*	segment 4	5	B
IX	Seg1-A8*	Segment 1	1.7	B
	Seg2-A8*	Segment 2	1.7	B
	Seg3-A8*	Segment 3	1.7	B
	Seg4-A8*	Segment 4	1.7	B
	Seg5-A8*	Segment 5	1.7	B

**Table 3:** Mitochondrial DNA (mtDNA) quantitation values and degradation index values for one recent segmented hair (R7) purified using protocols A and B. mtGenomes: mtDNA genome.

Extract	Protocol A		Protocol B	
	mtGenomes/cm	Degradation Index	mtGenomes/cm	Degradation Index
Segment 1 (proximal end)	43,992	1.53	17,261	1.50
Segment 2	17,836	2.33	11,185	1.90
Segment 3	11,568	2.53	3497	2.87
Segment 4	8,034	2.96	1414	2.91
Segment 5 (distal end)	10,296	3.44	2802	3.69

**Table 4:** Sequencing statistics for recent hair R7* purified with protocol B. This mapping used build hg38 as a reference. nuDNA: nuclear DNA.

Extract	Average mtDNA Length (bp)	Average NuDNA Length (bp)	Number of mtDNA Reads	Number of NuDNA Reads	Percentage mtDNA/nuDNA
Seg1 (proximal)	168	81	4255	103,015	4.0/96.0
Seg2	130	43	2066	76,713	2.6/97.4
Seg3	94	42	1271	62,481	2.0/98.0
Seg4	96	37	586	67,519	0.9/99.1
Seg5 (distal)	91	39	738	69,935	1.0/99.0

**Table 5:** mtGenome coverage for small fragments in recent hair R7*. The asterisk denotes extraction using protocol B. This mapping used revised Cambridge Reference Sequence (rCRS) as a reference to avoid gaps due to short reads that could either map to the mtGenome or to the nuclear genome (pseudogenes).

Extract	Coverage of at Least 2 Reads	Coverage of at Least 5 Reads	Coverage of at Least 10 Reads	Average Coverage	Coverage Range
Seg1	99.94%	96.10%	80.16%	26x	1x–184x
Seg2	86.96%	56.30%	31.01%	7.82x	0–100x
Seg3	73.85%	37.80%	15.09%	5.36x	0–77x
Seg4	42.27%	15.36%	5.79%	2.6x	0–33x
Seg5	55.88%	20.76%	4.97%	2.74x	0–21x

**Table 6:** mtDNA quantitation values, degradation indexes, and coverage over the mtGenome for six single recent hairs.

Extract	mtGenomes/cm	Degradation Index	Coverage of at Least 2 Reads	Coverage of at Least 5 Reads	Coverage of at Least 10 Reads	Average Coverage	Coverage Range
R1	8190	2.22	100%	99%	84%	18x	2x–47x
R2	400	1.93	73%	55%	39%	10x	0–163x
R3	44,447	2.39	100%	98%	82%	16x	2x–69x
R4	49,647	3.74	100%	100%	100%	30x	8x–122x
R5	103,181	2.14	100%	100%	100%	88x	38x–153x
R6	29,240	5.86	100%	100%	100%	67x	8x–844x

**Table 7:** mtDNA quantitation values, degradation indexes, and coverage over the mtGenome for aged hairs. The asterisk denotes DNA extraction using protocol B. NR: no result.

Extract	mtGenomes/cm	Degradation Index	Coverage of at Least 2 Reads	Coverage of at Least 5 Reads	Coverage of at Least 10 Reads	Average Coverage	Coverage Range
A1	20	3	8.28%	1.99%	0%	0.4x	0–9x
A1*	100	NR	100%	100%	100%	92x	20x–247x
A2	67	4.35	36.54%	11.9%	2.12%	2x	0–20x
A2*	947	NR	100%	100%	100%	84x	19x–232x
A6*	95	NR	100%	99.9%	98.98%	27x	3x–79x
A7*	2784	1.97	100%	100%	98.93%	27x	6x–715x

**Table 8:** mtDNA quantitation values and degradation Index values for two single aged segmented hairs. The asterisk denotes DNA extraction using protocol B. No degradation index was obtained for any of the samples.

Samples	Segment	mtGenomes/cm
A4*	Seg1	3330
	Seg2	2900
	Seg3	1950
	Seg4	510
A8*	Seg1	2401
	Seg2	952
	Seg3	678
	Seg4	379
	Seg5	49

**Table 9:** Sequencing statistics for single aged hairs. The asterisk denotes DNA extraction using protocol B [26].

Extract	Average mtDNA Length (bp; Ref hg38)	Average mtDNA Length (bp; Ref rCRS)	Average nuDNA Length (bp; Ref hg38)	# of mtDNA Reads	# of nuDNA Reads	Percentage mtDNA/nuDNA Reads
A1	71	65	88	145	131,100	0.1/99.9
A1*	55	54	54	44,826	5,132,784	0.9/99.1
A2	87	78	58	548	449,782	0.1/99.9
A2*	70	68	57	31,762	14,926,177	0.2/99.8
A6*	58	57	55	12,291	1,718,590	0.7/99.3
A7*	80	79	49	9352	112,190	7.7/92.3

**Table 10:** mtGenome coverage of one segmented aged hair segments extracted with Protocol B.

Sample	Extract	Coverage of at Least 2 Reads	Coverage of at Least 5 Reads	Coverage of at Least 10 Reads	Average Coverage	Coverage Range
A4*	Seg1	90.69%	43.47%	7.94%	5x	0–28x
	Seg2	96.18%	66.56%	15.2%	6x	0–37x
	Seg3	90.18%	46.93%	4.62%	5x	0–25x
	Seg4	85.53%	40.22%	1.7%	4x	0–26x
A8*	Seg1	100%	99.05%	93.6%	22x	2x–70x
	Seg2	85.17%	45.54%	6.44%	5x	0–21x
	Seg3	80.12%	30.89%	2.22%	4x	0–14x
	Seg4	80.85%	30.48%	1.89%	4x	0–16x
	Seg5	27%	0.85%	0%	1x	0–7x

**Table 11:** Sequencing statistics for recent single hairs.

Extracts	# Unique Human Reads	# mtDNA Unique Reads	# Unique nuDNA Reads	% mtDNA/% nuDNA (bp)	% mtDNA/% nuDNA (Reads)
R1	34,909	1865	33,044	11.95/88.05	5.3/94.7
R2	327,165	1707	325,458	0.59/99.41	0.5/99.5
R3	42,308	2287	40,021	9.29/90.71	5.4/94.6
R4	196,737	4790	191,947	2.88/97.12	2.4/97.6
R5	94,969	10,997	83,972	14.08/85.92	11.6/88.4
R6	952,728	25,575	927,153	3.56/96.44	2.7/97.3

**Table 12:** Sequencing statistics for single aged hairs. The asterisk denotes DNA extraction using protocol B.

Extracts	# Unique mtDNA Reads	# Unique nuDNA Reads	Average mtDNA Size (bp)	Average nuDNA Size (bp)	% mtDNA/% nuDNA (Reads)
A1	145	131,100	71	88	0.1/99.9
A1*	44,826	5132,784	55	54	0.9/99.1
A2	548	449,782	87	58	0.1/99.9
A2*	31,762	14,926,177	69	57	0.2/99.8
A6*	12,291	1718,590	58	55	0.7/99.3
A7*	9352	112,190	80	49	7.7/92.3

**Table 13:** Sequencing statistics for two segmented aged hairs.

Sample	Segments	# Unique Human Reads	# Unique mtDNA Reads	# Unique nuDNA Reads	Average mtDNA Size (bp)	Average nuDNA Size (bp)	% mtDNA/% nuDNA (Reads)	% mtDNA/% nuDNA (bp)
A4*	Seg1	39,870	1700	38,170	81	49	4.3/95.7	6.87/93.13
	Seg2	57,462	2129	55,333	75	43	3.7/96.3	6.23/93.77
	Seg3	43,982	1851	42,131	70	45	4.2/95.8	6.42/93.58
	Seg4	46,163	1655	44,508	69	49	3.6/96.4	4.66/95.34
A8*	Seg1	74,918	6834	68,084	92	70	9.1/98.9	11.62/88.38
	Seg2	36,674	1482	35,192	87	62	4.0/96.0	5.59/94.41
	Seg3	20,649	1171	19,478	90	54	5.7/94.3	9.14/90.86
	Seg4	30,569	1148	29,421	86	62	3.8/96.2	5.18/94.82
	Seg5	17,092	339	16,753	81	52	1.99/98.01	3.07/96.93

## Supplementary Materials

The following are available online at http://www.mdpi.com/2073-4425/9/12/640/s1, Figure S1: Comparison between the control region and the mtGenome coverage; Table S1: Global mtDNA qPCR results; Table S2: Coverage of the mtGenome for recent and aged hairs and hair segments; Table S3: Sequencing statistics for all recent and aged hairs; Table S4: Difference between shotgun sequencing and sequencing of libraries enriched for mtDNA; Table S5: The derived and ancestral Y haplogroup diagnostic SNPs that were observed for Sample A2*. Protocols: description of protocols A, B and C; CLC Workflow: workflow used to map reads to the mtGenome.
