# Supplementary material for: Fragmented Nuclear DNA Is the Predominant Genetic Material in Human Hair Shafts
Source: Genes (Basel). 2018 Dec 18;9(12):640. doi: 10.3390/genes9120640 (PMC6316335; doi:10.3390/genes9120640)
Supplement: Supplementary file 1 [file genes-09-00640-s001.zip › genes-391466-supplementary-final/CLC Workflow.pdf]

CLC Genomics Workbench Workflow for mtGenome

Trim Sequences	
Trim adapter list	MiSeq Adapter Consensus 4_18_16-R_ 8BaseIndex
Use colorspace	false
Also search on reversed sequence	true
Ambiguous trim	true
Ambiguous limit	2
Quality limit	0.05
Quality trim	true
Remove 5' terminal nucleotides	false
Number of 5' terminal nucleotides	1
Remove 3' terminal nucleotides	false
Number of 3' terminal nucleotides	1
Maximum number of nucleotides in reads	1000000
Minimum number of nucleotides in reads	25
Discard short reads	false
Discard long reads	false

Map Reads to Reference	
References	NC_012920.1
Masking mode	No masking
Masking track	
Match score	1
Mismatch cost	2
Cost of insertions and deletions	Linear gap cost
Insertion cost	3
Deletion cost	3
Insertion open cost	6
Insertion extend cost	1
Deletion open cost	6
Deletion extend cost	1
Length fraction	0.85
Similarity fraction	0.97

Map Reads to Reference	
Global alignment	false
Color space alignment	true
Color error cost	3
Auto-detect paired distances	true
Non-specific match handling	Map randomly

Remove Duplicate Mapped Reads (Original name: Duplicate Mapped Reads Removal)	
Maximum representation of minority sequence (percent)	20.0

Create Detailed Mapping Report	
Long contigs threshold	10000
Short contigs threshold	200

Fixed Ploidy Variant Detection	
Ploidy	2
Required variant probability (%)	90.0
Ignore positions with coverage above	100000
Minimum coverage	5
Minimum count	2
Minimum frequency (%)	10.0
Restrict calling to target regions	
Ignore broken pairs	true
Ignore non-specific matches	No
Minimum read length	20
Base quality filter	true
Neighborhood radius	5
Minimum central quality	20
Minimum neighborhood quality	15
Read direction filter	false
Direction frequency (%)	5.0
Relative read direction filter	false
Significance (%)	1.0
Read position filter	false
Significance (%)	1.0
Remove pyro-error variants	false

Fixed Ploidy Variant Detection	
In homopolymer regions with minimum length	3
With frequency below	0.8
