# Supplementary material for: Fragmented Nuclear DNA Is the Predominant Genetic Material in Human Hair Shafts
Source: Genes (Basel). 2018 Dec 18;9(12):640. doi: 10.3390/genes9120640 (PMC6316335; doi:10.3390/genes9120640)
Supplement: Supplementary file 1 [file genes-09-00640-s001.zip › genes-391466-supplementary-final/Protocols.pdf]

Supplementary Material

Fragmented Nuclear DNA is the Predominant Genetic Material in Human Hair Shafts

by Brandhagen et al.

Protocol used to wash and lyse/digest hair shafts.

Equipment/Materials

- General laboratory supplies (e.g., tubes, pipettes, vortex, forceps)
- Magnetic stand
- Stereomicroscope (optional)
- Sonicator

Reagents

- Xylene and/or xylene substitute, if needed
- Terg-a-zyme, powder or 5% solution (Alconox)
- Ethanol (EtOH), absolute
- Buffer ATL (Qiagen)
- Dithiothreitol (DTT), 5M solution
- Proteinase K (ProK), 20mg/mL
- Buffer AL (Qiagen)
- PrepFiler® Forensic DNA Extraction Kit (Thermo Fisher Scientific; cat# 4463351)
  PrepFiler® Magnetic Particles
  PrepFiler® Wash Buffers A and B
  PrepFiler® Elution Buffer (or TE^{-4} Buffer)
- Isopropanol, 70% Water, reagent grade or equivalent

Do NOT UV the Magnetic Particles or Proteinase K.

- Any step involving manipulations of difficult hairs may occur outside of hood with aid of stereomicroscope. Reverse action forceps may aid in grasping a hair. <u>The hood air flow must be off while transferring/handling hair samples.</u>
- View hair under stereomicroscope for presence of root tissue or adherent material.
- Measure hair and record length. Remove ~ 2 cm (or more if needed) of hair from root end and place in a tube.
  The sample may be moistened with water to minimize the effects of static.

Each wash procedure may be performed additional times using fresh cleaning solution.
Pulse spin, as necessary, throughout procedure to force sample to bottom of tube. A sample may remain in the same tube for each cleaning procedure with the removal of the cleaning liquid(s). In instances with smaller samples, smaller rinse tubes and/or less liquid may be used.

Xylene Wash (Optional)-Not used in this study.

- In chemical fume hood, add enough xylene to cover sample. Sonicate at least 20 minutes in chemical fume hood.

Alternatively, xylene may be added to the collection tube prior to the addition of sample.

- Remove xylene and appropriately discard waste. Add enough reagent grade water to cover sample and mix.

As an alternative to the xylene removal, sample may be transferred from xylene tube to a separate water tube with use of tweezers.

Xylene Substitute Wash (Optional)- Not used in this study.

- Transfer sample to tube containing enough UV-treated xylene substitute to cover sample. Sonicate at least 20 minutes.

Terg-a-zyme Wash

- To make 5% Terg-a-zyme solution, add 0.5 g Terg-a-zyme to 10 mL water.
- Transfer sample to tube containing enough 5% Terg-a-zyme solution to cover sample. Sonicate at least 20 minutes. Repeat once or twice if necessary.
- Transfer sample in a tube containing enough EtOH to cover sample and mix to rinse.

Transfer sample in a tube containing enough DNA-free water to cover sample and mix to rinse.

Lysis/Digestion

- To make the digestion Buffer (made fresh daily): add 13.2 µL 5M DTT to 1 mL Buffer ATL or 0.051 g DTT in 5 ml Buffer ATL.
- Add 300 µl of Digestion Buffer and 20 µL ProK to each sample and RB tube. Ensure samples are submerged. *Samples may be cut into pieces to ensure full immersion.*
- Vortex and incubate tubes at 56°C at 900 rpm for a minimum of ~30 minutes, until the sample is fully digested, or overnight (O/N).

Sample is generally transferred from the last cleaning tube to a new tube containing the digestion buffer; however, the sample may remain in the same tube with the removal of the water rinse prior to the addition of the digestion buffer.

If full digestion does not occur after a minimum of 2 hours, a partially-digested hair sample (and RB) may undergo the grinding process at the end of this procedure.

Purification protocol A: This protocol currently used by the DNA casework section at the FBI Laboratory. Favors recovery of larger (>100bp) DNA fragments

- Pulse spin tubes with lysate. Add 300 µL Buffer AL. Vortex tubes and incubate at 70°C at 900 rpm for 10 minutes.
- Pulse spin and allow to come to room temperature (~5 minutes).

PrepFiler® DNA Extraction Kit Purification

- Prior to addition, vortex PrepFiler® Magnetic Particles tube for 5 seconds until no visible pellet remains in bottom of tube. Pulse spin. *If processing multiple samples, vortex every ~5 minutes.*
- Add 15 µL of Magnetic Particles. Vortex at **low speed** for 10 seconds. Pulse spin.
- Add 180 µL of isopropanol. Vortex at **low speed** for 5 seconds.
- Mix at room temperature at 1,000 rpm for 10 minutes in shaker. Vortex at **high speed** for 10 seconds. Pulse spin.
- Place tubes in magnetic stand. Wait until size of pellet on back of tubes stops increasing (~3 minutes). With tubes in magnetic stand, pipette off and discard all liquid without disturbing pellet.
- Add 600 µL Wash Buffer A.
  Vortex at **high speed** until there is no visible pellet on side of tube (~5 seconds). Pulse spin.

It is acceptable to have visible aggregates in solution or on side of tube below meniscus.

- Place tubes in magnetic stand for ~60 seconds. With tubes in magnetic stand, pipette off and discard all liquid without disturbing pellet.
- Add 300 µL Wash Buffer A. Vortex at **high speed** until there is no visible pellet on side of tube (~5 seconds). Pulse spin.

It is acceptable to have visible aggregates in solution or on side of tube below meniscus.

- Place tubes in magnetic stand for ~60 seconds. With tubes in magnetic stand, pipette off and discard all liquid without disturbing pellet.
- Add 300 µL Wash Buffer B. Vortex at **high speed** until there is no visible pellet on side of tube (~5 seconds). Pulse spin.

It is acceptable to have visible aggregates in solution or on side of tube below meniscus.

- Place tubes in magnetic stand for ~60 seconds. With tubes in magnetic stand, pipette off and discard all liquid without disturbing pellet. With tubes remaining in magnetic stand, open and air-dry in hood with blower for ~8 minutes.

DO NOT OVERDRY.

If the room temperature is >25°C, reduce the drying time to 5 minutes.

- Add 50-65µL of Elution Buffer or TE^{-4}. Vortex at **high speed** until there is no visible pellet on side of tube (~5 seconds). Pulse spin.

- Incubate at 70°C and 900 rpm for 5 minutes. Vortex at **high speed** until there is no visible pellet on side of tube (~2 seconds). Pulse spin.
- Place tubes in magnetic stand. Wait until size of pellet on back of tubes stops increasing (~2 minutes.). Transfer liquid into final extract tube without disturbing pellet.

If an extract is discolored, spin 10,000 x g for 7 minutes and transfer supernatant to new tube.

Purification protocol B: uses a buffer that favors the binding of small (<100bp) DNA fragments to silica.

- To make binding buffer (for 5 samples), mix 16 ml PB buffer (Qiagen), 480 μl sodium acetate (3M) and 40 μl of sodium chloride (5M).
- Transfer the 320 μl lysate solution to a 15 ml conical tube and add 3 ml of binding buffer. Incubate on a flat shaker for at least 1 hour at room temperature.
- Prepare the Qiagen vacuum manifold by adding in order: one VacConnector (Qiagen, cat# 19407), one MinElute column (MinElute PCR purification kit, Qiagen) and one 3 ml extender (Qiagen, cat# 19587). Save the reservoir tubes provided with the columns.
- Transfer the binding buffer+lysate in the extender/column
- Wrap extenders loosely with para film to protect from contamination. Vacuum slowly until the MinElute column is empty.
- Add 3ml PE buffer (Qiagen) to the extender/column and vacuum slowly until the MinElute column is empty.
- Transfer the MinElute column back into the reservoir tube and centrifuge the columns for 1 minute at maximum speed.
- Transfer the column to a DNA free low binding 1.5 ml tube.
- Add 50-100 μl of Qiagen EB buffer or EBT buffer (EB buffer + 0.05% Tween 20) and let it stand for 5 minutes at room temperature.
- Centrifuge the columns for 1 minute at maximum speed.
- Pipette out the EB buffer and transfer it back into the same column. Let it stand for 5 minutes.
- Centrifuge for 1 minute at maximum speed. Remove column, close tube and store extract at -20°C.

Purification protocol C: produces two extracts per sample. One containing mostly the large DNA fragments lost Using Protocol B alone and one containing the small DNA fragments that can be lost using protocol A alone.

- Follow protocol A until section highlighted in red
- Place tubes in magnetic stand for ~60 seconds. With tubes in magnetic stand, pipette off and **TRANSFER** all liquid to a 15 ml conical tube, without disturbing pellet instead of discarding it.
- Mix the “liquid waste” with 5 ml of binding buffer described in protocol B.
- Incubate the solution on a flat shaker for at least 1 hour at room temperature.
- During this incubation, finish your first extraction following protocol A (wash the beads with Wash Buffer A, etc..).

→ Results in Extract # 1

2nd extract obtained with from the mixing of the liquid waste from protocol A and the binding buffer of protocol B as stated above (Bullet 3, Protocol C)

- Prepare the Qiagen vacuum manifold by adding in order: one VacConnector (Qiagen, cat# 19407), one MinElute column (MinElute PCR purification kit, Qiagen) and one 3 ml extender (Qiagen, cat# 19587). Save the reservoir tubes provided with the columns.

- Transfer the binding buffer+ lysate in the extender/column
- Wrap extenders loosely with para film to protect from contamination. Vacuum slowly until the MinElute column is empty.
- Add 3ml PE buffer (Qiagen) to the extender/column and vacuum slowly until the MinElute column is empty.
- Transfer the MinElute column back into the reservoir tube and centrifuge the columns for 1 minute at maximum speed.
- Transfer the column to a DNA free low binding 1.5 ml tube.
- Add 50-100 µl of Qiagen EB buffer or EBT buffer (EB buffer + 0.05% Tween 20) and let it stand for 5 minutes at room temperature.
- Centrifuge the columns for 1 minute at maximum speed.
- Pipette out the EB buffer and transfer it back into the same column. Let it stand for 5 minutes.
- Centrifuge for 1 minute at maximum speed. Remove column, close tube and store extract at -20°C.

→ Results in Extract # 2
