# Supplementary figures and images for: Fragmented Nuclear DNA Is the Predominant Genetic Material in Human Hair Shafts
Source: Genes (Basel). 2018 Dec 18;9(12):640. doi: 10.3390/genes9120640 (PMC6316335; doi:10.3390/genes9120640)

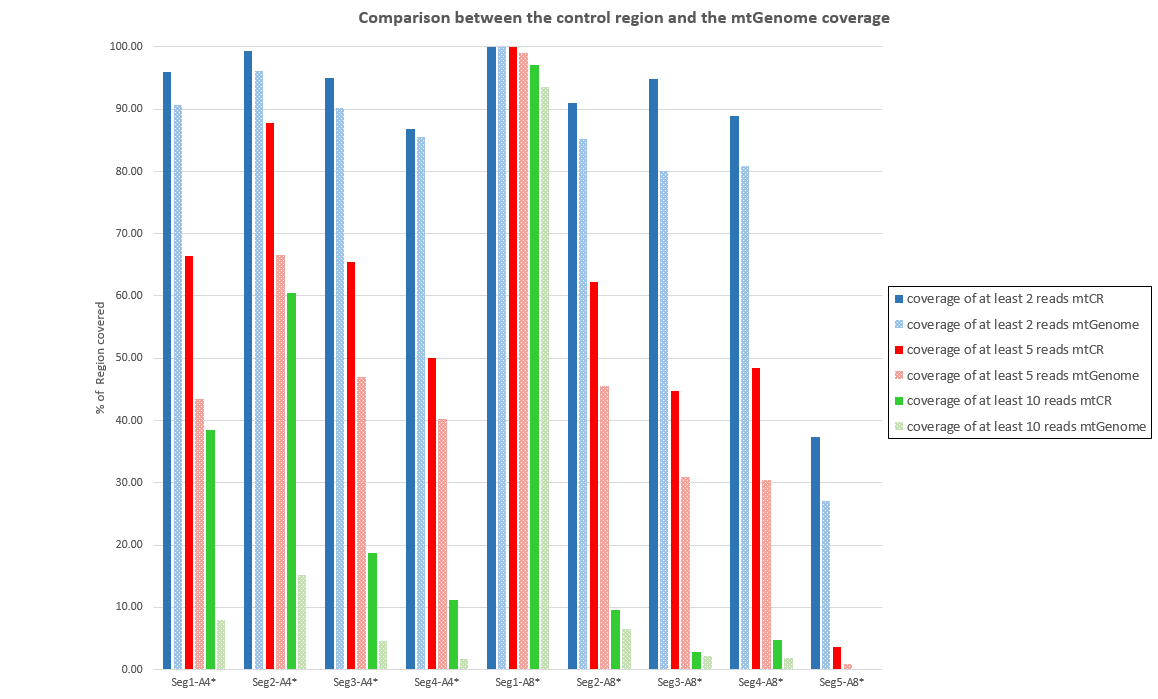

Supplement: Supplementary file 1 [file genes-09-00640-s001.zip › genes-391466-supplementary-final/Figure S1.PNG]
